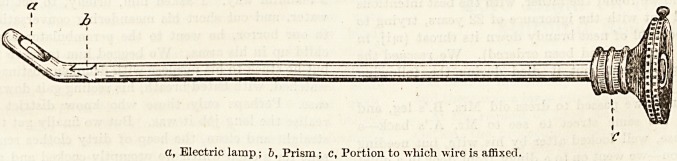# "The Hospital" Nursing Section

**Published:** 1906-06-23

**Authors:** 


### The Hospital.

### Nursing Section.

**Contributions for this Section of "The Hospital" should be addressed to the Editor, "The Hospital" Nursing Section, 28 & 29 Southampton Street, Strand, London, W.C.**

No. 1,031.—Vol. XL. SATURDAY, JUNE 23, 1906.

### Notes on News from the Nursing World.

### THE ARMY SERVICE AND EFFICIENCY.

An article in the current number of the "Broad Arrow" is apparently intended as an elaborate attack on Queen Alexandra's Imperial Military Nursing Service, but it seems to us that the points which the writer thinks he has made do not in the least reflect either on the system or its administration. For example, economy is insisted upon as "the order of the day," but the guiding principle of the Service has always very properly been efficiency, and it is significant that if, as our contemporary alleges, the nursing arrangements in military hospitals do not give satisfaction to the army surgeons, the only cause of their dissatisfaction is that the total of female nurses is not sufficient. As to the allegation that there is "discontentment among the lady nurses themselves," it is obviously open to those who are not contented to resign, but we have not heard complaints, either on the ground that they dislike the work of instructing the orderlies or any other. The answer to the assertion that the rank and file of the Royal Army Medical Corps object to the "disciplinary and other control placed in the hands of nursing ladies" is, that no disciplinary control whatever is exercised by the sisters. Their control is entirely moral, and, as a matter of fact, it has had a most excellent effect. Another assertion is that candidates for the Service do not come forward in adequate numbers, whereas the difficulty lately has been to select the most suitable applicants. Further, "the great and urgent need of men being trained during time of peace in nursing in our military hospitals," is recognised by the Army Nursing Board, who, on the showing of the "Broad Arrow" itself, have now 800 men being trained as nurses pure and simple. Traversing the more general statements of our contemporary, we have every reason to believe that most Army surgeons are emphatically of opinion that since the formation of Queen Alexandra's Nursing Service and the employment of educated women as nurses, the military hospitals have made remarkable strides forward, and that the main purpose in view, efficiency, has been achieved.

### THE COLONIAL NURSING ASSOCIATION STAFF.

The report of the Colonial Nursing Association, which was adopted at the annual meeting last week, shows that the organisation founded by Lady Piggott continues to make progress. The number of nurses at work during the year was 144, 108 being employed by the Government and 36 as private nurses, an increase of 23 upon the previous twelve months. Sir Frederick Hodgson, speaking at the meeting held in the Colonial Office, urged that when the nurses had gone through their terms of probation successfully and were willing to prolong their stay, they should be placed in the same position as Government officials of Colonies, and be put on the fixed establishment. We think that the suggestion is an admirable one, and we hope that Lord Elgin will be able to see his way to carry it out. As Lord Ampthill says, there are few better ways of showing interest in the Empire than by assisting the work of this Association; and one mode of attracting the most suitable women to its service would be the improvement of the position of the nurses.

### EXONERATION OF THE QUEEN'S NURSE.

Last week we were able to contradict the impression erroneously conveyed by a report in the daily Press of the alleged refusal of milk by a Queen's nurse at Holywood, Belfast, to a sick child because it was illegitimate, that there is any regulation in the rules of Queen Victoria's Jubilee Institute which would lead to such an action. We now learn with much satisfaction that the superintendent of the Irish branch, having fully inquired into the matter, is in a position to completely exonerate the nurse in question. It appears that instead of having refused to obtain nourishment for the child, the nurse had on a former occasion, when in attendance, recommended and obtained relief which was continued for more than a fortnight. Subsequently, being called to the case a second time, she found that the mother was neglecting the child, and she was then the means of having it removed to the hospital where it died. We also understand that it is inaccurate, as reported in the daily Press, that at the Coroner's inquest the nurse refused to say whether there was or was not a rule against recommending relief in such cases. Her statement was that the granting of relief rested with the Committee of her Association and not with herself personally. But the fact that relief had already been granted proved that the Association has no rule of the kind suggested. The exoneration is complete, and the vindication of the Queen's nurse from a charge which suggested inhumanity is a matter for congratulation to all concerned.

### DUTY AND DISCIPLINE.

The rumour of the compulsory retirement of two nurses belonging to Kingston Poor-law Infirmary caused a slight stir in the town and much regret, it being well known that the relations existing between the matron and her staff have always been particularly happy. The facts are as follows:—At the request of the Infirmary Committee the matron gave special instructions to nurses in charge of wards to be specially vigilant in watching the patients who were nearing the end of life's journey. The very next day, on returning to duty after dinner, three nurses left the ward unattended in order to make themselves tea. The matron happened to enter the ward in their absence and reported the matter to the medical officer. It was decided to separate the three nurses and change their charges. Two of them declined to accept this moderate form of punishment, or to apologise, and they were consequently suspended, but prior to the matter going before the whole Board, another endeavour was made to bring them to reason, which was not, however, successful. Under these circumstances, the Guardians, who might have dismissed them, pursued the lenient course of requesting them to resign, treating them also most generously in other respects. We cannot understand why these nurses should have hesitated to express regret at an obvious failure of duty, and, in the face of their refusal, it was absolutely impossible to retain their services and maintain discipline.

### LOCAL PROBATIONERS.

At the monthly meeting of the authorities of the Cameron Hospital, West Hartlepool, a report from the House Committee was read to the effect that after careful consideration of the question they had decided that it was not desirable that local persons should be appointed as probationers. Two speakers having urged on the contrary that local applicants should be given a chance as well as strangers, the medical officer of health supported the recommendation of the House Committee. He contended that the main trouble with the nursing staff in small towns had arisen through local probationers. They constantly asked, and got their friends to ask, for special leave; they talked a great deal about the work; and many of them indulged in tittle-tattle which gave rise to much mischief and ill-feeling against the institution. It was, however, determined not to bar applications from young women residing in the town or locality. We think that this is a wise decision. There is force in the objections of the medical officer of health, although there is at least one hospital in a town smaller than Hartlepool where the matron prefers local probationers, as she says that they settle down more readily, and instead of spending their money at places of amusement, can visit their own homes. But the proper course is to consider each application on its merits, whether from the locality or from a distance.

### THE LOCAL GOVERNMENT BOARD AND TRIVIAL DISPUTES.

When there are disputes between the master and matron of a workhouse on the one hand, and the nurses on the other, it is usually wise policy on the part of Guardians to suggest an inquiry by the Local Government Board. This has been done in the case of certain complaints made by two of the nurses in the sick wards of Carnarvon Workhouse. The complaints have been already investigated by a Committee of the Guardians, as well as the statements of the master and matron in reply, but in view of their conflicting nature, the Guardians considered it better to ask for the intervention of the department. There has also been a dispute at Cardiff Poor-law Infirmary; but in this instance the issues appear to be so small, involving only differences of opinion on the part of the superintendent nurse and a charge nurse on personal questions, that it may be hoped that the Chairman and Deputy Chairman to whom they have been remitted will be able to dispose of them. While the Local Government Board is the proper tribunal if principles are at stake, or a scandal has arisen, trivial matters should not be referred to it.

### QUEEN'S NURSES AND THE RATES.

We notice that it has been necessary for the manager of the Victoria Sick Poor Nurses' Association at Huddersfield to write to the local papers to correct an impression amongst the working classes in that town that the organisation is supported from the rates. As the Association is affiliated to Queen Victoria's Jubilee Institute, it may be worth while for us to state that in no case are any of the Queen's nurses supported out of the rates. Like the hospitals, they depend entirely for their maintenance upon voluntary contributions. If it had been otherwise, the Hospital Sunday Fund would not this year have decided for the first time to make a grant to the institution in London which sends Queen's nurses to the sick poor in their own homes, and thus relieves the strain upon the hospitals. In like manner Guardians frequently subscribe a sum of money to a nursing organisation in the provinces simply because they save the pockets of the ratepayers by diminishing the number of inmates in the Poor-law Infirmary, or the sick wards of the workhouse.

### AN AUSTRALIAN MATRON AND HER NURSES.

The difficulties experienced by some institutions in this country in securing good nurses are also met with at the Antipodes. The matron of the Perth Public Hospital in a report to the Board of Management, states that it is not so much the number as the quality of the nurses which is making the work so hard for individual members. She receives plenty of applicants for the position of probationers, but she finds it far from easy to fill satisfactorily the vacancies caused by the resignations of staff nurses. Within twelve months she lost six, who either left to take up higher posts or to go to the Eastern States to obtain special certificates. Like matrons in England, she also finds that it is not every nurse who gets her certificate who is competent to manage a ward.

### IMPERIAL MILITARY NURSING SERVICE.

We are officially informed that Miss M. H. Congleton, Miss C. H. E. Gerahty, and Miss A. M. S. Clapp have been appointed staff nurses in Queen Alexandra's Imperial Military Nursing Service. Miss J. E. Dods, sister, has been transferred to the Royal Victoria Hospital, Netley, from the Military Hospital, Gosport; Miss J. W. Wilson, to the Military Hospital, Gosport, from the Royal Victoria Hospital, Netley; Miss M. R. Makepeace, to the Military Hospital, Cork, from the Royal Victoria Hospital, Netley; Miss E. Beck to the Military

Hospital, Pretoria, from the Military Hospital, Middelburg, Transvaal; Miss C. Anderson to the Military Hospital, Middelburg, Transvaal, from the Military Hospital, Potchefstroom; and Miss D. M. Taylor, to the Military Hospital, Cairo, Egypt, on arrival from England. Miss A. R. Sibbald, staff nurse, has been transferred to the Cambridge Hospital, Aldershot, on appointment. Miss G. E. Saunder, matron, and Miss E. S. Mason, sister, have been instructed to hold themselves in readiness for service abroad.

### THE HEYWOOD-JOHNSTONE MEMORIAL HOME.

Just a year has elapsed since the foundation of the Heywood-Johnstone Memorial Home for Trained District Midwives, in the Old Kent Road, and the event was celebrated by a pleasant little meeting on Thursday last week. Dr. Handfield Jones took the chair, and Dr. Champneys showed his interest in the Home by his presence. The speeches were few and short, for the chief interest of the entertainment centred in the "judging" of 125 babies, all born under the care of the midwives and pupils of the establishment, by Dr. Lionel Smith, assisted by the matron of the York Road Lying-in Hospital. The Hon. Mrs. Talbot presented prizes to the best babies, who were arranged in classes according to age. The mothers then had tea in the garden, and the visitors were also regaled in the house. The length of training for the midwives is four or six months. Six midwives have been trained during the year, who all successfully passed the examination of the Central Midwives Board. Two hundred poor women were attended in their own homes, and there was no fatal cases recorded among the mothers, whilst only four babies were either born dead or died.

### TRAINING MIDWIVES IN MONMOUTHSHIRE.

We are indebted to the superintendent of the Monmouthshire Training Centre for Midwives, Sister Barrett, for the first report of an undertaking that promises to be of the greatest value to the county. Started only on January 2, 1905, the training school has supplied such a want that ten nurses were trained in practical work last year, and six which now hold the certificate of the Central Midwives Board. For the first few months the training was carried on at the hospital, but it then became necessary to secure a Maternity Nurses' Home. As the result of a series of addresses at mothers' meetings, the money was obtained, and a suitable Home with telephonic accommodation was opened in May. The number of cases attended from February 17 to December 31 was 236, the number booked being 303. The nurses paid 3,588 visits to these patients, and the superintendent 708. A medical man was called in 24 times, the calls being divided among 14 doctors. There was but one death; and no case either of puerperal fever or of hæmorrhage occurred. Thirteen children were still-born, but none died in the birth. When the pupils have finished their training they are at liberty to do monthly nursing, taking their own fees. A pleasing feature is that the movement has been generously supported by the ladies of Monmouthshire, who, however, speaking through the committee, affirm that the untiring efforts of the superintendent to train her pupils, and at the same time benefit the district, are beyond all praise, and that to her most of the credit for the success of the undertaking is due.

### THE DISTRICT NURSE AND THE CARE OF INFANTS.

At the National Conference on Infantile Mortality last week, Dr. Niven, of Manchester, classifying the educational agencies specially dealing with the care of the infant, referred to the Midwives Act as offering "a prospect of getting young mothers warned and instructed in the procedures needful to protect their offspring before and after birth, especially if trained and experienced nurses become midwives." He mentioned the fact that an example of the possible extension of the sphere of nurses had already been provided by Manchester; the Education Authority, pending the requisite special training of the school teachers, having appointed Miss Grace Taylor, who has been trained as a nurse, to give a course of instruction in the care of infants to older girls in the schools. As to this educational work, he said that the only fault that he had to find was that there was not enough of it, but the difficulty lay in getting teachers with the necessary training and experience. He thought that it was doubtful whether anyone who has not had experience in the management of infants in hospital could profitably give such a series of lectures as those given by Miss Taylor. For this subject, hospital or other equivalent training would, he was convinced, be required.

### AN UNREGISTERED MIDWIFE ACCUSED OF MURDER.

At the South-Western Police Court on Tuesday, Amelia Hollis, aged 51, described as an unregistered midwife, was charged with the wilful murder of the wife of a baker carrying on business in Wandsworth. Her arrest followed the close of the Coroner's inquiry into the circumstances of the death. It was stated that the prisoner attended the deceased and that her death was due to an illegal operation performed by the midwife. The latter denied the charge, and she was remanded.

### SHORT ITEMS.

The King has approved of the appointments of Miss Emily Du Pre, Mrs. Teresa Ellen Richardson, Mrs. Ellen Bertha Tufnell, and Mrs. Elizabeth Ann Macpherson as ladies of grace of the Order of the Hospital of St. John of Jerusalem in England.—The Nurses' Home and Laundry at the North Eastern Hospital for Children, Hackney Road, Bethnal Green, will be opened by the Lord Mayor of London on Monday, July 2, at 3 p.m.—The first annual conversazione of the Provisional Committee of the National Council of Nurses was held on Thursday night last week at the galleries of the Society of British Artists, the hostesses including Mrs. Bedford Fenwick, Miss Isla Stewart, matron of St. Bartholomew's Hospital, and Miss Barton, matron of Chelsea Poor-law Infirmary.—In connection with the addition of a new operating theatre and the completion of other improvements at Bourton-on-the-Water Cottage Hospital, a dedicatory service was recently held, followed by a tour of inspection by a number of gratified visitors.

### The Nursing Outlook.

"From magnanimity, all fears above;
From nobler recompense, above applause,
Which owes to man's short outlook all its charm."

### A GREAT NURSE-TRAINING SCHOOL.

### II.—Developments of Earlier Years.

One great function of the training school is to keep in touch with every old worker who has been trained within its walls. There can be no doubt whatever that the influence upon character of the ties which bind individuals to their alma mater are of the strongest and best. It is therefore matron's custom to be at home on one week-day evening to all old workers, and to make her annual letter so full as to keep each of them informed and up to date as to the changes and improvements which each year has brought to the work and all connected with it. The establishment of a supplementary register in 1893 has kept the matron well informed of the work and movements of most certificated nurses from the dates of their leaving the service of the hospital. Of course this register is dependent upon the co-operation of the old workers, but it is pleasant to note that the large majority of them have recognised the practical value of this system, and that it has been possible to make it successful during the years which have intervened since the date of its establishment. Old workers are encouraged to write every six months, and in any case to make it a rule to celebrate their birthday as a nurse by writing on that date each year with the latest account of themselves to their old matron. Miss Lückes' system has been from the first to keep in the closest possible touch with every nurse who has passed through the London Hospital school, and she has always made it her business to be available to every one of them should they desire to consult her at any time when in a difficulty. In this way the tone and character of the army of London Hospital nurses who have continued in the profession has been maintained at a high level of excellence.

It is interesting too to notice certain developments in the school with their dates. Thus Tredegar House (1895), which followed the lines of Miss Strong's Glasgow scheme, has softened the ordeal to probationers of entering hospital life. It has saved many unsuitable candidates from the annoyance of a failure in hospital work, whilst it has economised the patience and time of the sisters and staff nurses in the hospital to an extent which is greatly appreciated by them. Tredegar House may be regarded as the sieve of the nursing school. Once in seven weeks twenty selected candidates are received into Tredegar House, where their suitability for the work is thoroughly tested by careful preparation and thorough instruction. Those who show aptitude are then admitted to the wards as probationers where the effect of the preliminary training is exhibited by the improved quality of the service they are able to render in the wards, under the sisters' and charge nurses' direction. Looking back it is not easy to realise how in the earlier days of training it was possible for a sister to make headway when she might have to depend largely on the health of untried novices who had neither gifts nor real inclination for nursing work. That is why the institution of Tredegar House marks an epoch in the development of this school.

In 1894 an important step was taken by the appointment of an independent examiner charged with the duty of setting the papers and conducting the examinations of all candidates for certificates in nursing. This change added immeasurably to the value of the certificate, and it was at once recognised as evidence of the thoroughness of Miss Lückes' method. Then followed the introduction of models and many useful appliances to secure that the bandaging and other classes should be thoroughly practical. The introduction of systematic instruction by means of cookery classes under special arrangement with the National Training School of Cookery, which has been followed by several important developments, was another and earlier feature of the school, which has had marked results for good in several directions. In 1899, thanks to Queen Victoria's liberality, the Finsen light was introduced into the electric department, which was placed upon a permanent footing. Not only has the London Hospital been able in consequence to treat one hundred cases a day, but it has been the means of training a goodly number of nurses who are now carrying on the work in other hospitals up and down the country. We have said enough to show that this training school has kept well to the front, and that so far as the training of nurses is concerned everything has been done to make it a practical success. There are several other matters yet to be dealt with which we must reserve until next week. It would be an education for many people to spend a couple of days in going thoroughly into the details of the nursing system and work at the London Hospital. We could wish that quite a number would so educate themselves without delay.

### The Care and Nursing of the Insane.

By Percy J. Baily, M.B., C.M.Edin., Medical Superintendent of Hanwell Asylum.

### I.—ANATOMY AND PHYSIOLOGY.

(*Continued from page* 161.)

### 7. The Excretory Organs.

We saw, when speaking of the digestive system, that in the production of energy—by which is meant heat, motion, thought, glandular secretion, etc., etc., the word energy being simply another term for the work done by an organ or tissue—the materials of which our tissues and organs are built are oxydised or slowly burned up, and as a result of this certain waste matters, comparable to the smoke and ashes of a furnace are produced. These waste matters are thrown by the organs which produce them into the blood, which acts as the general carrier in the economy of the body. But in order that health may be maintained the blood must be kept up to a certain standard of purity, and the waste matters must not be allowed to accumulate. The organs whose duty it is to remove these deleterious waste matters from the blood are spoken of as the *excretory organs.* They are the *lungs, kidneys,* and *skin.* We must now devote a little space to an inquiry into the nature of these waste matters, and of the arrangements with which we are provided to enable us to get rid of them.

Here it may be as well to explain the difference in the meaning of the terms *excretion* and *secretion.* Both are materials which are manufactured in the body, and both are the products of glands, but an *excretion* is a product which has no further use within the body—something which must be got rid of as speedily as possible, such, for instance, as the urine and the sweat—whereas a *secretion* is a glandular product which is capable of doing useful work within the body, which is, in fact, essential to the maintenance of life, such, for example, as the saliva, the gastric juice, or the pancreatic juice. But although one never speaks of the " excretion " of saliva or gastric juice, it is not uncommon to speak of the " secretion " of urine or sweat.

Nature is in the habit of economising, and hence we find that few of our organs have only one office or function to perform. Of the excretory organs the lungs and skin are no exception to this rule; they have other business to attend to over and above their excretory function. The kidneys, however, are exceptions, since their whole attention is occupied with the manufacture and excretion of urine.

#### A.—*The Respiratory System.*

We already know that the blood which circulates in the systemic arteries (arterial blood) differs remarkably in appearance from that which flows through the systemic veins (venous blood), and we have seen that this difference in appearance is dependent upon the relative amount of oxygen gas contained in these two varieties of blood—arterial blood being rich in oxygen, whereas venous blood, although still containing some of this gas, is relatively deficient in it. At the same time we know that venous blood contains far more carbonic acid gas than does arterial blood.

It follows, then, that at some part of the circulation these two gases—oxygen and carbonic acid—must in the one case enter, and in the other leave, the blood, and from what we have learned already when studying the circulation, we know that this occurs while the blood is passing through the capillaries. This *interchange of gases* on the one hand *between the blood and the atmosphere* (that is, in the pulmonary capillaries), and on the other *between the blood and the living tissues* (that is, in the systemic capillaries) constitutes the *Function* of *Respiration.*

Before we can properly understand the process of respiration and the mechanism whereby we breathe, it is necessary to know something of the physical properties of the gaseous envelope which surrounds the whole world, and which we call the atmosphere. It is composed of a mixture of certain gases, and by its weight exercises a considerable pressure upon the surface of the globe, which may be looked upon as the bottom of a vast ocean of air. We may say that it is somewhere about 40 miles deep, and that beyond this, out into the unfathomable abyss of space, there is nothing but the theoretical ether. We human beings are therefore living at the bottom of this vast ocean which presses upon us with an enormous weight (equal to 15 lbs. on every square inch of the body's surface); but as this weight is equally distributed all over our bodies it is not appreciated by us.

Since, however, the pressure of the atmosphere is so great, it follows that every hollow space which is not occupied by some more tangible material—some solid or fluid substance—must be filled with air, or otherwise it would be crushed in. In other words, when we say that a box or a bottle is empty we really mean that it is filled only with air—it must contain air, since a vacuum or space which does not contain even air does not exist in nature, and can only with great difficulty be produced artificially.

As to the chemical composition of the atmosphere, it is sufficient for our purpose here to say that it is a mixture of three gases, of which one only is all important. This is *Oxygen.* It is for the purpose of getting oxygen into our bodies that we breathe. We cannot exist without oxygen. It is through the chemical union of oxygen with the elements of the tissues of our bodies, and of the food we eat (especially, though not only, the carbon), that all forms of energy or work are produced, and the same chemical process—oxydation—is the origin of heat. When a fire burns in the grate the heat which is produced is the result of oxydation of the coal. In like manner the heat of our bodies is the result of oxydation of the materials of which our tissues are composed, and the whole object of the function of respiration is to provide our tissue elements—the cells which in countless myriads build up our bodies —with oxygen, and to remove from them the carbonic acid which results from the oxydation of the carbon which exists in them. But if we breathed pure undiluted oxygen, the materials of our tissues would very soon be burned away; we should, in fact, live too fast. This is prevented because oxygen in the air is diluted with another gas which chemically is quite inactive. This second gas is called *Nitrogen.* It exists in the atmosphere apparently simply for the purpose of diluting the oxygen than which it is found in much larger quantities. Roughly, there is rather more than three and a half times as much nitrogen in the air as oxygen.

When dealing with the subject of alimentation and food we saw that our tissues are composed of certain chemical elements, of which the chief are oxygen, hydrogen, carbon, and nitrogen, and we learned then that these elements must be present in the food to supply the tissue waste which life entails, and to build up our bodies in the process of growth. Now, although all classes of food contain oxygen, we require so much of this element that it is quite impossible to meet the demands upon it by the food alone, and so we take it in its free state from the atmosphere. It might at first be thought that in like manner the demands for the supply of nitrogen could also in part be met in this way since with every volume of oxygen we inhale, we draw into our lungs more than three times the amount of nitrogen; but this is not so. Free nitrogen—that is to say, nitrogen not in chemical combination with some other chemical element—cannot be dealt with by our tissues in the process of assimilation, and the whole of the nitrogen required must be supplied in the form of nitrogenous food.

The third gas which is found in the air is carbonic acid. This in pure fresh air, such as is found among the mountains and at sea, exists only in very minute quantities, but in cities, where the air is smoky, or in buildings where many people are congregated, such as in church or the theatre, it is much more prevalent. In a hundred volumes of pure air—say a hundred bushels or a hundred pints or a hundred cubic feet—about 21 of these volumes would represent the amount of oxygen, and the other 79 would be nitrogen, whereas the amount of carbonic acid would only be 1-25th of a volume, or, stated in decimals, .04. Hence in speaking of the quantity of these gases in the air, we say that 21 per cent. is oxygen, 79 per cent. nitrogen (roughly), and .04 per cent. is carbonic acid.

### The Nurses' Clinic.

### CYSTOSCOPY.

The cystoscope is an instrument by which the interior of the bladder is illuminated, enabling the surgeon to inspect the organ, and in case of doubtful diagnosis determine definitely the nature of the disease from which the patient suffers. By its aid ulcers of the wall of the bladder, visual calculi, and cystitis can all be seen and diagnosed, but one of its most important uses is to ascertain, in cases of renal disease, whether one ureter and kidney is acting normally before operating upon or removing the diseased one. For this purpose the catheter cystoscope is used; a long fine gum-elastic catheter, made specially for the purpose, is passed along a tunnel at the side of the cystoscope and introduced into the ureter, the surgeon guiding it by means of the cystoscope, and the urine is drawn off direct from the kidney into a sterilised test-tube without coming in contact with the bladder.

*a*, Electric lamp; *b*, Prism; *c*, Portion to which wire is affixed.

For cystoscopic examination the patient should be prepared by having an aperient about midday the day before, an enema simply during the evening to ensure the bowels being freely moved, a warm bath at bedtime, and on the morning of examination a wash-out enema. A light breakfast should be given early, followed by a pint of weak tea half an hour before the time fixed for examination, after which the patient should be requested to refrain from passing urine if possible.

Should the urine be turbid, however, as in cystitis, or there be hæmorrhage into bladder, it will be necessary to have bladder washed out before proceeding with the cystoscopy. This is generally done by an assistant a short time before the arrival of the surgeon; he will require a catheter, gum-elastic or rubber, with funnel end; a little sterilised oil, a syringe, and a jugful of either warm boracic lotion or plain warm water; also a receptacle for urine drawn off.

The surgeon will require sterilised towels and gauze, two sterilised gallipots (one containing sterilised oil and one sterilised glycerine), a long narrow specimen glass or similar receptacle three parts filled with 1 in 20 carbolic, in which the cystoscope should be placed lamp downwards (taking care to keep the sterile end dry, or otherwise the instrument will be spoiled), half an hour before the examination; rubber and gum-elastic catheters (several sizes), with funnel ends; a syringe, a test-tube, a piece of rubber tubing, a large jug of warm boracic lotion; a bottle of adrenalin in case of hæmorrhage into bladder, the blood making fluid cloudy and rendering examination futile; a firm pillow or sandbag, to raise the patient's pelvis; dilators of different sizes (for male patients), and a receptacle for fluid drawn off.

The patient should wear warm stockings reaching well above the knees and a short flannel or gamgee jacket, in addition to the usual blanket over chest.

Care should be taken to keep the patient warm on being returned to bed, male cases being liable to rigors, especially if dilators have been used, in which case it is well to have quin. sulph. and tinct. opii handy, these being given as routine treatment by some surgeons. Immediately chloroform sickness has passed off patient should be kept quietly in bed for the remainder of the day.

The nurse should be careful not to boil any gum-elastic

**THE NURSES' CLINIC—*Continued.***

catheters, as boiling causes the surface to blister and renders them useless. They should be immersed in 1 in 20 carbolic, and immediately before use placed in plain warm sterilised water. Many surgeons bring their own electric battery, but should the cystoscope be connected with main supply it sometimes happens that there is a leakage of electricity, owing to the apparatus for reducing the voltage being overtaxed. In such case it will be wise (if a metal operating table be used and rubber mats are not available) to place a bath mat or old blanket thickly folded under feet of table, as otherwise anyone touching it may receive a very unpleasant electric shock.

### Incidents in a Sister's Life.

### A DAY IN THE DISTRICTS.

Doubtless to all of us at some time or other in our lives some echo of the world's doings sets up a vibration in the chords of memory which may, perchance, be growing rusty, and we "fight our battles o'er again" with the keen enthusiasm of youth quickening our slower pulse and stirring our stiffened joints. Sitting here at my quiet desk, the news of the Jubilee of the "Biblewomen and Nurses Mission" calls to mind some vivid pictures of past days—days of hard work, strain, and anxiety, yet full of absorbing interest, and not without touches of humour among the pathos, which otherwise must have filled our lives with sadness.

How well I remember starting out early one morning,—we had a small operation on in one of the districts at 9.30 A.M.—thinking perhaps that I might be able to finish up in good time and get home to tea. Arriving at nurse's rooms at 9 (an hour earlier than usual), the first thing was the official duty of going through the case-book and cupboard, etc., and then we proceeded together to the operation case. It was only the removal of a cyst just above the eye in a baby of 16 months, and nurse had seen to the preparation of the room the night before. So we had everything in readiness by the time the doctor came, and as he had no assistant with him, I took charge of the chloroform, holding the artery-clips in the other hand, while nurse looked after the swabs and needles.

Our next case was a new confinement—twins, born the night before. They were first babies, so the one room which formed the dwelling still bore indications of brand-new furniture, with gay window-curtains which had already had their day. One of the twins was a very poor little specimen, and we found the father, with the best intentions in the world but with the ignorance of 22 years, trying to pour a teaspoonful of neat brandy down its throat (♏ij. in ʒj. of water was what had been ordered). We rescued the baby for the moment, but it died during the following night.

Looking in as we passed to dress old Mrs. B.'s leg, and stopping in the same street to see to Mr. A.'s back—a paralysed case, well looked after by his wife, but needing our inspection—we went on to a dingy front door in a quiet street, and mounted the stairs to a poor but neat room, where, in a wicker chair near the window, sat a sweet-faced old woman. This was one of those slow cases of cancer of the breast, which heal up for weeks at a time and then break out in fresh hæmorrhage, each a little worse than the last. The trembling old fingers were doing some patchwork with bits of silk we had given her, but it was quickly put aside, and I only crossed the room just in time to gently prevent her rising to greet me. The wound was now healing, and I knew that nurse dressed it with unfailing regularity and efficiency, but old Miss C. always felt happier if "Sister" did but look at it on her weekly visit. So the bandages were taken off, though not before the dear old lady had insisted on performing her usual ritual of placing a clean towel on the footstool in front of her for me to kneel on! Truly, as I knelt there smiling, with a hot feeling at the back of my eyes, I always felt that she was the benefactor, with her sweet brave face and patient eyes, that looked so trustingly forward. When she was worse and we had her in bed, white and weak from loss of blood, she would still smile and say, "It's one step nearer home; they've all gone there before me, you see."

Then there was a little hip-disease boy—an out-patient of Guy's, who went up to the hospital once a month for inspection. His tubes were to be taken out and washed and the wounds redressed; and I had to call in to see Mr. H. (just recovering from rheumatic fever) about getting him away to our Convalescent Home. And by this time we wanted some lunch; so, to save time, we turned into a shop for coffee and sandwiches.

Afterwards we made our way to Mrs. D., whose husband was caretaker of a club chiefly used by working men with a turn for politics. This poor little woman was in rapid consumption, and after her first reserve had broken down we learnt that she had made a foolish marriage with a boy three years younger than herself, and who was only now twenty! There was a two-year-old babe, who looked only half that age. When we reached the room, which was at the top of the big, empty house, the sound of a sob reached us outside the door. Pushing it open we beheld our patient sitting up, with a dishevelled air, crying bitterly, and the boy-husband stretched across the bed, dressed, in a heavy drunken sleep. In the perambulator on the other side of the room, out of reach of the helpless mother, the wailing child lay on a sodden heap of clothes. The room was in an abject state of neglect, dirt and untidiness, permeated by a strong smell of stale beer and whisky. The man woke up as I put my arm round the weeping woman, and began to apologise in a maudlin way. I asked him, briefly, to get us some hot water, and cut short his meandering conversation. Then, to our horror, he went to the perambulator and took the child up in his arms. We begged him to leave it with us, but he stuck to his purpose with drunken obstinacy, and we watched, with bated breath, his reeling gait down the staircase. Perhaps only those who know district work will realise the long job it was. But we finally got things a bit straight and clean, the heap of dirty clothes removed, and sent for a chop which we promptly cooked and gave to the patient; she had not tasted any food that day, and the baby had only had a drop of stale milk. I ventured downstairs to ask for the child again, and this time was allowed to take it, giving the poor mite in its turn a warm bath and dry clothes and food, while nurse went off to find a neighbour to come in and do some washing.

There were still a few cases to see after that, and when they were done I looked at my watch and found it was nearly six, and I had to go to another district half an hour's journey off to see a bad typhoid case whom I visited every day to make arrangements for the night nursing.

It was only an ordinary day's work, but it so filled up every nook and cranny of one's existence that there was no room left for anything but sleep, as soon as one's head touched the pillow—that long, deep sleep that brings us back in het morning to fresh life, with renewed vigour and hope to go forth to our duties again.

### The Government and Registration.

### PROPOSED OFFICIAL DIRECTORY OF NURSES.

A deputation, organised by the Central Hospital Council for London, waited upon the Earl of Crewe, Lord President of the Council, on Thursday last, in order to present a scheme of an official directory of nurses as a practical alternative to State registration, and to protest against the Bill now before Parliament. The deputation was received at the Privy Council Office. The names of the deputation were announced last week.

Mr. H. A. Harben, Chairman of the Council, dealing with a memorandum embodying the proposals, explained that it represented eighteen of the largest hospitals in London, which employed a nursing staff of 2,200 and turned out five or six hundred nurses every year. He proposed the establishment of an official directory as an alternative to State registration. As a necessary consequence of registration, all those nurses not upon the register would have to be regarded as inefficient and improper persons to whom to entrust the care of the sick. It should be remembered, however, that as a rule a nurse was employed not by the patient but by the medical man, who knew her capacity and efficiency and whether she was suitable for the precise kind of work required of her. There were numbers of imperfectly trained nurses who were well fitted for many branches of their work, and at the same time many highly certificated nurses who did bad work. Registration meant a continuous guarantee by the State of a nurse's fitness and efficiency. Once registered, it would be almost impossible to remove a nurse's name from the list unless she were guilty of some criminal offence.

The Hon. Sydney Holland expressed confidence in Lord Crewe's sympathy on the ground that he was a trustee of the Nightingale Fund, and ventured to ask him to consult Miss Nightingale before coming to a final conclusion on a subject that had been so long agitating the nursing world. There had been a preponderance of evidence in favour of State registration before the Select Committee of the House of Commons, but that was because its opponents were disinclined to produce adverse testimony over and over again. State registration, in its refusal to admit uncertified nurses, would cast a slur upon women who were doing excellent work in the country. Certain heads of large hospitals—the matrons of St. Bartholomew's and the London—for example—had not had the training necessary to admit them to the contemplated State register. Even Miss Nightingale would not be qualified, "and yet," Mr. Holland added, ironically, "I believe she has done some good work in the nursing profession." Even if a Central Board for the purpose of carrying out State registration were possible, there was no constituency to elect it except, perhaps, a few retired matrons who had nothing better to do than to agitate up and down the country. The effect of an official directory would be to stimulate public inquiry into the question of what training a nurse had had; but if, on the contrary, they had a State register, people would imagine that no further inquiry was necessary. Registration would be detrimental to advance, for it would set up a standard of minimum efficiency and tend to stereotype mediocrity.

Sir Thomas Barlow thought that registration would dislocate the relations between medical men, the nurses, and the public. He believed that if a poll were taken of medical men their chief complaint would not be in regard to the inefficiency of nurses, but in regard to discipline and conduct. In the doctor's absence, and especially on night duty, a nurse's conduct was sometimes tyrannous and even cruel. The desirable thing was to maintain the relations between a nurse and her training hospital, and a system should be organised by which she might return to the wards, thereby learning up-to-date methods and keeping in touch with the matron. He regarded the use of the term "profession" by nurses as a disastrous one, tending to make them independent of medical men and matrons. At the same time he fully sympathised with the feeling that their work should be properly recognised, and he considered that the directory under discussion would effect this purpose. It would contain no delusive statements, but would set forth what the nurse had learned and the details of her experience. The proposed official directory would be a valuable security in the case of nursing homes, many of which were run on a purely mercantile basis.

Lord Crewe, in the course of his reply, recognised that the deputation represented a large volume of public feeling. He could not help thinking it strange that there should be such a marked difference of opinion on this matter, for he knew it would not be disputed that among his hearers' opponents there were some whose views could not be ignored. The conclusions of the Select Committee of the House of Commons must have due weight, and he supposed that as an abstract proposition a system of registration was desirable if it inflicted no injury on existing nurses. He thought Mr. Harben scarcely fair in declaring that nurses not upon the register would have to bear the stigma of inefficiency. He saw little or no analogy between the registration of medical men and that of nurses. It was impossible for the Government to take up the question this Session, nor could he make any promise as to next Session. But meanwhile it was desirable that there should be as much discussion as possible. A Bill on the subject of registration was coming on shortly, and he suggested to the deputation that a Bill embodying their views in the same amount of detail should be introduced into Parliament. The House of Commons had little time to spare at present, but the Upper Chamber was open to discussions on questions of abstract character.

Mr. Harben, in thanking Lord Crewe for his courteous reception, intimated that his suggestion would be placed before the Central Hospital Council.

### Lord Elgin on the Colonial Nursing Association.

The annual meeting of the Colonial Nursing Association was held in the Library of the Colonial Office on June 13th, and there was a large attendance.

At the request of Lord Ampthill, President of the Association, the proceedings were opened by Lord Elgin, who said that he had the honour of being the first Secretary of State who had the pleasure of welcoming the Association to a meeting held at the Colonial Office. Although that was the case, he thought that the relations which had subsisted between the department and the Association had throughout been of the most cordial and intimate character. It was about ten years ago, in June 1896, when his predecessor, Mr. Chamberlain, sent a prospectus of this Association to the Colonies in a circular despatch, with a strong recommendation of its objects; and seven years later, in 1903, in a very important despatch, which went into the whole subject of tropical diseases, Mr. Chamberlain again dwelt on the importance of nursing and of the value of the work which this Association was doing. He considered that it was worth while reminding the Association of the fact of those references to their work in important despatches, as it showed that it had been the object of the Colonial Office to regard the provision of nurses as part and parcel of its general endeavour to improve the conditions of health in the colonies under their management. When they remembered that they had colonies in almost every part of the habitable globe, and in many countries where the conditions of life were unfavourable to the health of white people, they would recognise the inestimable boon which trained nursing must be, and, therefore, the debt which the country owed to the Association for endeavouring to assist in providing such nursing in the Colonies. He believed that the result of these references in the despatches had been that considerable interest had been taken in the Colonies themselves and by the governors in authority in the Colonies in the provision of nurses. It was an enterprise in which they had to enlist the co-operation and sympathy of ladies, and the movement had had from the beginning the great advantage of the interest of Mrs. Chamberlain. The Colonial Office had been represented constantly on the committee, and he wished to express the thanks of the office for the services of Lady Ommanney and Mrs. Antrobus, who had represented them. He had not the means of offering any suggestions as to the work of the Association, but he signified the interest which was officially taken in its existence and operations, and he hoped that the proceedings that day would be taken as a visible sign and token of that interest.

Lord Ampthill moved the adoption of the report, which was taken as read, and said that it was a very satisfactory record of progress and improvement in every direction. There was, however, one aspect of it which was not altogether satisfactory, and that was that their funds were not increasing as they desired. There was not a time when sympathy was more needed than when a public servant of the Crown in one of those distant Colonies was struck down by illness; it was then that a courageous Englishwoman, going out as a nurse, interpreted the national sympathy. There were few better ways of showing interest in the Empire than by assisting the work of this Association.

Sir Frederick Hodgson, in seconding the adoption of the report, said that there was one point to which no reference had been made in it. He thought that when nurses had gone through their term of probation successfully, and were willing to prolong their stay, they should be placed in the same position as Government officials of Colonies, and be put on the fixed establishment, enabling them to retain a pension when they retired. In that way governors could better recognise the good work of the Association, and he would do his utmost to make some arrangement of this kind in his Colony. Speaking from personal experience, as formerly Governor of the Gold Coast, and now in Demerara, he testified to the excellent and invaluable work done by skilled nurses in those parts, where there was already a drop in the death-rate as a result of their labours.

### The Promotion of the Higher Training of Midwives.

### ANNUAL MEETING AT LONDON HOUSE.

The annual meeting of the Council for the Promotion of the Higher Training of Midwives took place at London House on June 13, Princess Christian, President of the Council, in the chair.

Miss Alice Gregory, Hon. Secretary, opened the proceedings by describing the work and aims of the training school founded by the Council, the "Home for Mothers and Babies," at Woolwich. Referring to the general result of the Midwives Act, Miss Gregory instanced some of the difficulties incident to so great a social reform, but asserted that despite many discouragements there were plenty of signs of healthy life, and with a little more impetus the results of the year's work should be in enormously increasing ratio. London was at the head of the good work. The London County Council were touching a higher level than anything yet attempted by any public body in offering twelve annual scholarships, all for a course of six months' training, to women of sufficient education to pass the sixth standard, preference being shown to candidates with some training in general nursing. But London was a land apart, and one's heart went out to the country and little country towns; for the old "Gamps" were dying out, pushed out, frightened out, and after 1910 the shortage would be far more seriously felt. They must, anyhow, be thankful that the women in London would some day be properly looked after. This was especially where the Council felt its mission lay. It aspired to found a training school where educated women could be trained in all the many qualities that went to the making of a district midwife. To observe closely, to handle delicately, to diagnose correctly, to obey faithfully, to attain that measure of self-control which would make them a refuge and support to those in the "Valley of the Shadow of Death," such was their ideal. But day by day the conviction grew that they would never touch the fringe of what was to be done until they could unite a general with the maternity hospital. Educated gentlewomen would never give themselves in any numbers to this work until they could be offered a complete training school under one organisation, and to solve the national problem satisfactorily they must secure and train those women who were now often seeking worthy employment up and down the country, or entering professions already overcrowded. Moreover, our large general hospitals fought shy of admitting a patient who was near her confinement, and the maternity hospitals, on the other hand, refused to take in cases complicated with any serious complaint, so these poor women were practically outside hospital aid. Was not that alone a sufficient reason for the existence of an institution where the patient could be instantly carried from a maternity to a general block, or *vice versa*? Several hospitals had started a maternity ward of a few beds to meet this need; and what they had done on a very small scale the Council desired to do on a large one. The Home for Mothers and Babies at Woolwich was designed to accommodate six mothers and to train two pupils at a time. These numbers had rapidly grown to fifteen mothers and four pupils, and they had to constantly refuse applications for want of room and funds. In conclusion, Miss Gregory appealed earnestly for help to meet these great needs—the needs of the nation.

Mr. W. Crooks, M.P. for Woolwich, spoke heartily of the excellent work done by the Home, and advised all who wanted to do something, or to spend their money, to send their names to Miss Gregory.

Dr. Cullingworth, referring to the opening of the Home last year by Princess Christian, said he must confess that, interesting and valuable as the present hospital was, and strong as its claims to support were on account of the local benefits that it confers, its chief attraction from his point of view was that it was the earliest and at present the only embodiment of a great and worthy aspiration. The Central Midwives Board had now established a State examination, and issued rules and regulations, making it impossible for women to become enrolled as midwives without proof of a certain standard of education and training. But this standard required to be supplemented by a long course of special training in the functions they have to perform for a midwife to be the potent instrument for civilisation which she ought to be, and which the scheme they were there to advocate that day is endeavouring to secure that she should become. Moreover, in encouraging educated women to take up district midwifery Miss Gregory was not carried away by false sentiment and mistaken philanthropy. On the contrary, she was able from her own personal experience of the needs and the difficulties, to train the pupils for district work, and to teach them how they may become the accepted advisers of the poorer classes, diffusing an atmosphere of cleanliness, cheerfulness, and thrift, unsullied by the dust-cloud of condescension or the mist of a sham philanthropy.

Sir John B. Riddell then proposed a vote of thanks, which was seconded by Dr. Mary Rocke, who spoke in warm terms of the work of the Home and the training in infant care and management, which not only the midwife, but the mother, received during her fortnight's sojourn.

### London Biblewomen and Nurses Mission: The Jubilee.

A CROWDED meeting in honour of the jubilee of the London Biblewomen and Nurses Mission was held at the Œlian Hall on Monday last.

At the back of the platform were ranged a number of nurses, who sang "Now thank we all our God" at the beginning of the meeting with very good effect.

The chair was taken by the Marquis of Northampton, who remarked that he felt great pleasure at presiding at the inaugural jubilee meeting of this Mission, which deserved the sympathy and support of all those living in this great city. After a brief survey of the events of the past fifty years he said that he himself had had personal experience of the Nurses Mission in one district in London, and had monthly reports sent to him of the work, which made him feel more and more that it ought to be multiplied a thousandfold. It was a question of a Christian woman going to the bedside of the suffering, a woman who had been trained in the highest way, so that she knew what should be done, and was able to receive instructions of the medical man, and carry them out correctly, and thus greatly to assuage the suffering even of those afflicted with incurable diseases. He felt that if the public only knew the good results of this work they would come forward and help it far more in the future than in the past. He had just heard that the Jubilee Fund had reached the figure of £2,000, but he hoped that it would eventually be £4,000.

Lord Kinnaird (Treasurer) made a brief financial statement, which showed that in the past three years they had had an annual deficit of £2,000, which had been met by legacies. He thought that they were reasonable in making the request that the Jubilee Fund should be largely supported, so that in future they would be able to make both ends meet.

Sir Frederick Treves said that he was glad to be able to testify to the admirable work done by the Mission, looked at from a medical standpoint. There was an impression that the needs of the sick poor were entirely met by the hospitals. But all connected with hospitals could tell the same story, that the saddest tragedies were those that took place outside their walls. Every medical man had two pictures in his mind—that of the cases which came to the hospital too late, and that of the relapsed cases. Every year there went into the wards of the hospitals men, women, and children who had been allowed by neglect, largely due to ignorance, to get into such a condition as to render their state almost hopeless. Many of these cases would have been curable if treated soon enough. This Mission dealt with that class of cases. These admirable nurses found their way into all parts of this great city, and saw that such cases were properly attended to. Then came the relapsed cases which the nurses of this Mission helped; the treatment begun in hospital was continued, and the patient watched and assisted. If the work of the Mission could be multiplied by one hundred, relapsed cases should be unknown in the annals of the hospitals.

Miss Andrews, the Honorary Superintendent, in reviewing the history of the Mission, said that after the death of Mrs. Ranyard the Nurses Mission was lifted on to a much higher condition of efficiency, until to-day it would rank with all the other great nursing societies. They tried to keep the surgical nurses abreast of the modern discoveries. They encouraged nurses to go in for examinations, and were in touch with the hospitals, so that they knew when new methods were introduced. They were in close communication with the out-patient departments of many of the great hospitals, and the nurses received direct instructions from the doctors in attendance there, and were thus enabled to save poor mothers many a weary tramp to the hospital, since if a child had the attention of a skilled nurse, one visit a week to the hospital sufficed instead of two or three. They were also doing all in their power to combat the great scourge of tuberculosis and to impress upon the poor the imperative necessity for taking adequate precautions.

### Everybody's Opinion.

### ARE NURSES UNDERPAID?

"ARGYLE" writes: Will you kindly allow space in reference to the very excellent speech by Mr. Pollitt at the meeting of the Royal National Pension Fund on June 6? Mr. Pollitt has grasped the situation and gone straight to the point. He believes in educating the public to the idea of larger salaries for nurses. One hears so much of impressing on nurses the necessity to save. And why? Nurses are not thriftless as a class. A good nurse who has done work for twenty or thirty years ought to command a salary to enable her to make the future secure, and live not only independent but comfortable when she retires. At the present rate of remuneration a nurse is unable to do this. Many nurses give up a great deal to pay into the Pension Fund, and this for £15 or £21 at fifty-five. Now, a nurse may be able to do good work at fifty-five or she may not. How many have friends to go to? or, again, how many would care to be dependent on friends, and eke out an existence on £21 a year? It seems to me a very frail craft to embark on. I thank Mr. Pollitt most heartily for bringing this forward, as I, with many others, feel there is a call for considerable reform.

### IN DEFENCE OF FEVER NURSES.

"VETERAN" writes: A lot has been said and written recently about a much-maligned portion of our noble profession—namely, fever nurses. A plea for them is an urgent necessity. Without going into details as to the wisdom of compulsory removal, isolation hospitals, or massing of infectious diseases generally, it is sufficient to say that the need has been "created," and the work is as important in every sense of the word as the most brilliant surgical triumph. Many a loved darling of an ignorant or drunken parent owes its life and health to the care bestowed on it during its temporary residence in fever hospitals. Then why all this outcry against them? The Metropolitan Asylums Board employs three years certificated nurses in charge of all their wards, and in almost every instance rural district councils do the same. Yet that this special branch of our work is despised is noteworthy. I think that the remedy is not far to seek. Let all who undertake it resolve to win the respect and admiration of their subordinates by showing them what a hospital-trained nurse's status really is. Charity suffereth long and is kind, and the motto may well be adopted—To speak no evil.

### Appointments.

Central London Ophthalmic Hospital.—Miss Stella Weston has been appointed matron. She was trained at the London Hospital, and has since been sister at the Royal Hospital for Incurables, Putney; out-patient sister at the Royal London Ophthalmic Hospital; and assistant matron at the City of London Hospital for Diseases of the Chest.

City Hospital, Lodge Moor, Sheffield.—Miss K. Jarvis has been appointed staff nurse. She was trained at Park Hill Hospital, Dingle, Liverpool, and has since been staff nurse at the City Hospital, Netherfield Road, Liverpool. She has since done private nursing in Southport.

General Hospital, Stourbridge.—Miss Ada Morton has been appointed staff nurse. She was trained at the Royal Albert Hospital, Devonport.

King's School Infirmary, Canterbury.—Miss B. X. Hall has been appointed nurse in charge. She was trained at the Royal Infirmary, Sheffield, where she was afterwards sister. She has since been nurse at the Samaritan Free Hospital, London; charge nurse under the Metropolitan Asylums Board; nurse in charge of the Infirmary of the Royal Masonic School for Boys, Wood Green; and nurse in charge of the Infirmary of the Royal Naval School, Eltham.

Medical Mission Hospital, Plaistow.—Miss A. E. Elliott has been appointed staff nurse. She was trained at Whipps Cross Infirmary, Leytonstone, and was afterwards charge nurse at West Ham Schools Infirmary. She has since been charge nurse at the Dauns Schools, Sutton.

Queen Victoria Memorial Hospital, Welwyn.—Miss M. Snow has been appointed matron. She was trained at the Middlesex Hospital, and has since been Queen's nurse. She holds the certificate of the Central Midwives Board.

Royal Victoria Infirmary, Newcastle-on-Tyne.—Miss Edith McCall Anderson has been appointed matron. For upwards of three years she has been assistant matron of St. George's Hospital, London.

Salisbury Isolation Hospital.—Miss Margaret Brown has been appointed matron. She was trained at the Royal Infirmary, Dundee, and has since been night superintendent at the General Infirmary, Salisbury. She holds the certificate of the Central Midwives Board.

Salop Infirmary, Shrewsbury.—Miss F. M. Long has been appointed night superintendent and Miss M. H. Allibone sister. Miss Long was trained at the Royal Hants County Hospital, Winchester, where she was afterwards sister. She has since been matron of Basingstoke Cottage Hospital. Miss Allibone was trained, and has been staff nurse, at Shoreditch Infirmary. She has since been sister at Shirley Warren Infirmary, Southampton.

St. Mary (Islington) Infirmary.—Miss Hilda Hyliffe has been appointed night superintendent, Miss Florence Fox sister, and Miss Emily Parker staff nurse. Miss Hyliffe was trained at Birmingham Infirmary, and has since been sister at Portsmouth Infirmary and sister at St. Mary (Islington) Infirmary. Miss Fox was trained at St. Mary (Islington) Infirmary, where she has since been staff nurse. Miss Parker was trained at St. Mary (Islington) Infirmary.

### Novelties for Nurses.

(By our Shopping Correspondent.)

### AERTEX CELLULAR CLOTHING.

The arrival of hot weather will cause nurses who have prudently been awaiting a rise in the thermometer to change to cooler clothing to at once look out for lighter garments. For this purpose they could not do better than visit Messrs. Oliver Bros.' establishment at 417 Oxford Street, London. From personal experience in South Africa and in the tropics I can vouch for the wonderful coolness and comfort of the Aertex cellular clothing. This particular kind of fabric has many claims upon popularity, the chief being that it is cool in summer and warm in winter, is easily washed and does not shrink, is very durable and does not irritate the most sensitive skin. Blouses made in this material are delightfully cool, and possess the additional advantage of requiring neither starching nor getting up. Messrs. Oliver make them from 7s. 11d., the "cut" is very good, there is a detachable turn-over collar, and the colours are attractive. Dressing jackets, from 5s. 11d., look most inviting; corsets, which start from 3s. 11d., if once worn, will be continued; and nightdresses, from 4s. 11d., in very light materials, should be most delightful on airless nights. There is, in short, every single article of underclothing to be had in this material, including vests, bodices, combinations (from 3s. 11d.), and petticoats. All garments can be made to order at an additional cost of 1s. each.

### TRAVEL NOTES AND QUERIES.

By our Travel Correspondent.

Tour for a Month in Brittany (Queen's Nurse).—You do not say what you can afford, so I am quite in the dark. Most convents object to big boys, but I am giving you the names of some, to which you can write on a prepaid foreign return letter-card, asking the question, and also inquiring terms. Some of these religious houses are now possibly closed. Maison Ste Anne at St. Servan, Convent at St. Jacut de la mer, I have forgotten the exact name, but it is not essential. The cheapest spot I know is Lancieux. Write to Hôtel des Bains Lançieux par Ploubaley Côtes du Nord. Terms from 5 francs. Then there is Perrosguirec Hôtel de Levant and Hôtel des Bains and Paimpol Hôtel Continental; all these places are on the coast, and cheap.

To Speak French for Three Weeks (L. R.).—I know of no French family who would take you on your terms; at your highest figure it only means a fraction over 6 francs per day. I should advise you to go to some cheap hotel in Brittany, where every one will speak French, though it may not be perfect. See addresses given to "Queen's Nurse." Normandy is too expensive, and you would hear more English.

Wales or Somerset (C. B.).—At Barmouth write to Proprietor of "Marine Mansion," terms from 30s. weekly; or to "Glencairn." Barmouth is in a very attractive neighbourhood. An easy excursion is the Cader Idris tour, and there are endless beautiful walks round. I should consider it a better holiday ground than Minehead. I have no addresses at this place, but you can have them by writing to the Traffic Manager at Paddington, enclosing stamp, and asking him to send you their list of lodgings, farmhouses, etc. Excursions from Minehead, Watchet, Dunster, Lynton, and Lynmouth.

Address in Malvern (Nurse F.).—I have received the following address, which may suit you: Mrs. Burlingham, 1 Birchwood Villa, Albert Park Road. Terms from 25s. weekly, or if two friends share one room 21s. weekly.

Bracing Air in Cornwall or Devon (Swansea).—This can only be found in the North. I know of no rooms, but you can be comfortably boarded at extremely reasonable terms with Miss Snell, Ivy Cottage, Caerhays, near St. Austell, Cornwall. There is no station, but St. Austell is not far off. You must remember that the presence of a station sends prices up at once. Another address is that of Mrs. Alan Murray, Brookside, Croyde, North Devon, reached easily from Braunton Station. How would you like Lynton or Lynmouth? It is now quite in the world reached by train from Barnstaple. Prices range much higher there; but I think it is more what you want. Write to the Traffic Manager at Waterloo and ask for the lodging and farmhouse list. Enclose stamp.

Hydro at Ilkley (M. C. B.).—Write and ask terms at the Hydro; it would not be dear on the third or fourth floor. Other addresses are "Wells House Hotel" and "Crescent Boarding House." You might also write, enclosing stamp, to the Traffic Manager at King's Cross, and ask him to send you their gazette with list of lodgings and farmhouses.

### A Book and its Story.

### THE TRAINING OF A GENIUS.*

Mrs. Fred Reynolds in "The Making of Michael" has produced with much artistic charm a very fascinating study of the boyhood and youth of a musical genius. The creation of Michael was a labour of love to the author, the reader must feel, for he is drawn with the gentle and unerring touch that insight, combined with sympathy, alone can give. No more picturesque description can be found in the annals of child life than that of the beautiful gifted boy, living under the charge of the warm-hearted, rugged-featured, north-countrywoman Nan, in the farmhouse on the fells. His coming to her has been marked by mystery, and no hint of his parentage has reached her. She had received, through a firm of solicitors, a handsome sum for his maintenance. So liberal, indeed, that there was a considerable balance remaining every year at the bank, which she placed to his credit for future needs. Nan herself, reticent and unattractive, has her dark life-tragedy to bear, and around the child Michael centres the only brightness in a life otherwise desolate. Michael has one other friend and director in the parish priest, Dr. John, who is also his tutor. A charming vignette of Michael is given on the opening page, "Beside the laughing stream played a little child. He was all alone, yet his clear, silvery voice rose ever and anon in babbling talk. Occasionally he would clap his hands with a sudden access of joy, and a peal of elfin laughter would mingle with the music of the stream. . . . Very happy and content was he in his apparent loneliness. So much so that he never perceived a stranger had invaded his solitude. The stranger, having an artistic eye, paused in admiration; paused, too, being a woman, in wonderment to see so dainty a child in such an unfrequented place. . . . Who was this beautiful, lonely child? She felt sure he was beautiful, though as yet, beyond a smooth curve of sun-flushed cheek, she had not seen his face; but hands and legs were delicately shaped, and the glimpse of neck, between the silky mop of hair and the top button of the little smock, was so smooth and choicely modelled she longed to have the right to kiss it." After a long and remarkable dialogue between the stranger and the child, in which the evidence of the artist nature is apparent in every question of his, in his disappointment at finding a camera which she is carrying is only after all a thing that takes photos, and does not, according to his ideas, make pictures, the stranger finds she is losing caste with the child, when, in reply to many inquiries as to what a photo really is, she is obliged to confess that it makes pictures, but without colour. "There will be no colour in it." "What a pity," said the child, adding, with a little sigh, "It won't be so very much better than a photograph, then." "It will be a photograph." "You said it was a picture," returned he, rather reproachfully. "And is a photograph not a picture?" "I s'pose it might be called one," he answered, drawing gradually a little farther away. . . . There were a few moments' silence—a silence which seemed to knock at the heart of the woman, though the child looked not at her, but away into the distance, his dainty chin supported by one tiny hand. "Things with colour are more satisfying," he said dreamily. . . . Meanwhile, quite regardless of his companion, the child, accustomed evidently to express his thoughts aloud, continued: "Colour is the second best thing there is." "And what comes first?" . . . The child gave a little laugh. "Sound," he said, as though there could not be two opinions on that score. "Sound, the singing of things, of course. These really are the chief things, aren't they?" A request to be allowed to take a photo of the child is refused with politeness. "I think I'd rather not," he said, "if you don't mind." "But why?" "I don't think myself would like to be in that black box." "It wouldn't be yourself." "It would look like me." "Of course." "Then wouldn't it feel like me?" "Why, no!" "How can you tell it would only look like me and not feel like me, how can you?" Again no direct answer was forthcoming. In reply, when asked if his mother would not like a photo of him, he shakes his head negatively. "Won't you ask her?" "I can't." The stranger's eyes filled. "Is she dead?" she inquired gently. "Dead!" laughed the child, "my mother dead! Why, she is just the livest thing here, 'cept my father. . . . It is quite, quite true," chuckled the boy. Then reading displeasure in the face looking down into his, he explains that the earth is his mother and the sun his jolly, laughing father. "He is with me generally all day long; but my mother, my dear, dear mother," with a caressing nestle of his silky head among the moss and ferns, "she is with me always." When the stranger has recovered from the surprise caused by this announcement, she feels some definite information is imperative about the real parentage of the boy. Her efforts to gain this are not assisted by his reply to her question of "How old are you, boy?" . . . At this the child abandoned his leaning attitude, and drew himself straight up, his little hands finding their way to his breeches pockets. "Well!" he considered, his head slightly on one side, "I don't know 'zactly. I 'spect I'm a good age, fifty perhaps, or a hundred. I must be nearly that. I've known myself such a long, long time." The stranger hesitates to accompany the child home, but yields to his importunity. "I shall be dreff'ly disappointed," he said, "if you do not come." With the most graceful of childlike bows he held out his little hand. . . . The stranger took the proffered hand and went with the child. From his foster-mother, Nan, she learns the story of the child being found by her outside her door one Christmas Eve when she, a solitary, heart-broken woman, mourning the tragic death of her only child, at the hands of an insane father, stood listening to the church bells. She was suffering acutely. "The burden laid upon me seemed greater than I could bear, and I cried unto the Lord to give me deliverance from the agony of my grief. . . . I do not know how long I had been pouring out my soul before Him, when suddenly there came a great calm, and I knew it was night and very still. Then on the silence came, far off, the sound of bells. Joy bells they seemed to me. . . . Then came voices singing, singing—sure as I stood here alone in this room—'Unto us a child is born.' But all the while the bells sounded so loudly I could scarce hear the voices. So I thought I'll open the door and maybe I'll hear them more plainly." Then she discovers the child. "He was a fair babe to look upon, and always good and untroubled. . . . Gazing at the wonder and perfection of him, at times I almost dared to hope it was none other than the Christ child come on earth again." "Did you ever find out where he came from?" "To this day I do not know. . . . Twice a year since then the money has come, and with it the clothes. Ah! it is these that call the stubborn tears from my weary eyes—dainty clothes, sewn by a woman's hand. And who shall say that her eyes have not been dim as she sewed them for the child she never may see?" "The Making of Michael" is a unique book, charming alike in conception and in treatment.

* "The Making of Michael." By Mrs. Fred Reynolds. (George Allen, 6s.).

### Notes and Queries.

**REGULATIONS.**

**The Editor is always willing to answer in this column, without any fee, all reasonable questions, as soon as possible.**

**But the following rules must be carefully observed.**

1. **Every communication must be accompanied by the name and address of the writer.**
2. **The question must always bear upon nursing, directly or indirectly.**

**If an answer is required by letter a fee of half-a-crown must be enclosed with the note containing the inquiry.**

*Reading to the Sick.*

151) Will you kindly tell me the name of the book from which a quotation entitled "The Dove on the Cross" recently appeared in your columns.—*A Reader since* 1887.

"Rays of Sunset for Dark Days," price 3s. 6d., published by Messrs. Macmillan, and can be obtained from the Scientific Press, 29 Southampton Street, Strand.

*Queen's Nurse.*

(152) How can I become a Queen's nurse? Is 44 years of age too old?—*Whittaker.*

See "How to Become a Nurse," published by the Scientific Press, 28 and 29 Southampton Street, Strand, W.C.

*Chiropody.*

(153) Will you tell me the best place to learn chiropody?—*Lota.*

You might consult "The Practical Cure of Corns and Bunions," by E. Harding Freeland, F.R.C.S., published by John Bale, Sons, and Danielson, price 1s. It can be obtained from the Scientific Press, 28 and 29 Southampton Street, Strand, London, W.C.

*Workhouse Hours.*

(154) My work as night head lunatic attendant is very hard. Can you suggest anything I could do?—*Holloway.*

You might appeal to the Matron, and if she does nothing, go before the Guardians.

*Midwives and Doctors.*

(155) Is there any redress against small and cheap doctors running down, and warning patients against, fully-qualified midwives?—*Irate.*

Certainly you can obtain redress if a doctor or anyone else says anything libellous against you personally.

*A Nursing Home.*

(156) Can you advise a reliable nursing home in Brighton or London?—*May.*

We do not recommend private nursing homes. The Nurses' Co-operation, 8 New Cavendish Street, may suit you.

*Norland Institute.*

(157) Is the Norland Institute for trained nurses an institute which children's nurses can join?—*Inquirer.*

The Norland Institute trains children's nurses. For further particulars write to the institute.

*Arthritis.*

(158) Can you tell me where a respectable woman suffering from rheumatoid arthritis could be received?—*Penzance.*

If she is not bedridden write to the Sister-in-Charge, St. Peter's Home, Kilburn.

*Nursing Homes in India.*

(159) Can you give me the names of Nursing Homes in India?—*India.*

Lady Roberts has a home, but there are no vacancies, and there is the Up-Country Nursing Association. Address Mrs. Sheppard, 10 Chester Place, Regent's Park, N.W.

*Useful Information.*

(160) What can I do to prepare myself as a hospital nurse? *Omega.*

Study physiology and anatomy and sick cooking. Then write for a catalogue of Nursing Manuals from the Scientific Press, 28 Southampton Street, Strand.

*Recognised Training School.*

(161) Is the K—— Union Infirmary a recognised training school for nurses?—*W. W.*

Yes, certainly.

**Handbooks for Nurses.**

| | Post Free. |
|---|---|
| "A Handbook for Nurses." (Dr. J. K. Watson) ... | 5s. 4d. |
| "Nurses' Pronouncing Dictionary of Medical Terms" | 2s. 0d. |
| "Art of Massage." (Creighton Hale.) ... | 6s. 0d. |
| "Surgical Bandaging and Dressings." (Johnson Smith.) ... | 2s. 0d. |
| "Hints on Tropical Fevers." (Sister Pollard.) ... | 1s. 8d. |

Of all booksellers or of The Scientific Press, Limited, 28 & 29 Southampton Street, Str[illegible], London, W.C.

### For Reading to the Sick.

RETROSPECT.

Here, on the borders of that better land,
Shall pain's sharp ministry for ever cease;
Then shall we bless Thee safely landed there,
And know above how good Thy teachings were;
Then feel Thy keenest strokes to us in love were given,
That hearts most crushed on earth shall most rejoice in
Heaven.

*Anon.*

Let us see whether we cannot ourselves get something of the wider outlook of nature. There are those who wish for death, because life has been hard to them, because they are disappointed and wearied. There are those who, with brave and steadfast hearts, face the inevitable, with no clear conception of what it means, with no hope beyond the grave. But, oh! that we could learn with the Apostle more of the widened outlook of life beyond the grave, not as rendering this life poor and worthless, but as giving it its right meaning and orientation.

"To depart to be with Christ," that was his conception of death. A living Lord asks our love, that He may enrich and fulfil it in a life of power, in a life of wide and unbroken outlook, which shall look beyond prison walls of adverse circumstance, the sorrows and straits of life, and the fear and shrinking of death, to that city which hath foundations, whose Builder and Maker is God, "Where I shall at last know even as I am known."

The power of Christ's Resurrection, while it dignifies life here, while it strengthens the will, consecrates the body, and stimulates the whole moral and spiritual being, opens out the wide vision of eternal life, where beyond these voices there is peace.—*Canon Newbolt.*

The stars grow brighter as the night darkens. As the lights of earth are put out one by one, the countenance of heaven makes plainer revelations. Grace makes a very sunset of what to nature is the most impenetrable darkness, and the plaintive strains of the *Miserere* merge in spite of humility into songs of triumph; for the walls between the dying soul and the heavenly Jerusalem are so nearly fretted through that the loud Alleluias mingle with the contrite love whose eyes are closing on the Cross. Precious in the sight of the Lord is the death of His saints.—*F. W. Faber.*

. . . From the calm shores of the land of everlasting life have I watched thee, my beloved child, toiling through the waves of this troublesome world . . . and now, because the night draweth on apace, and the darkest hour is ever before the morning, I have come to thee upon the billows, that I may be near thee in thy time of peril; and behold, I am with thee in the ship! . . . Fear not; they who follow Me shall never walk in darkness; thy footsteps shall not slip; mercy shall hold thee up when perils encompass thee about; and though the sunshine of this world's joys be dim for thee, in My light shalt thou see light.—*The Divine Master.*

Guide, from Thy love's abundant source,
What yet remains of this day's course;
Help with Thy grace, through life's short day,
Our upward and our downward way;
And glorify for us the west,
When we shall sink to final rest.

*Wordsworth.*

## Figures and Tables

**Figure f1:**